# Mad Honey Disease: A Rare Condition in an Unlikely Locale

**DOI:** 10.1155/carm/2632633

**Published:** 2025-06-25

**Authors:** Sheikh W. Jamal, Eyad Elamir, Shybin Usman, Harris Poolakundan, Maryam Almahri, Adnan Abdul Khaleq, Zidan Darwish, Eithar Musa, Anas Zayad, Bassem Al Hariri

**Affiliations:** ^1^Department of Medicine, Hazm Mebaireek General Hospital, Hamad Medical Corporation, Doha, Qatar; ^2^College of Medicine, Qatar University, Doha, Qatar; ^3^College of Medicine, Weill Cornell Medicine-Qatar, Doha, Qatar; ^4^Medical Education Department, Hamad Medical Corporation, Doha, Qatar

**Keywords:** cardiotoxicity, honey, Nepal, Qatar

## Abstract

**Background and Aims/Introduction:** Mad honey disease is caused by consuming honey containing grayanotoxanes—neurotoxins found in certain species of *Rhododendron* plants. Mad honey, derived from the nectar of these plants, can cause significant cardiovascular and neurological symptoms. While most cases are reported in regions where it is produced, extensive travel among diverse expatriate communities in various global regions is one of the factors that may contribute to cases occurring in nonendemic areas. Other factors leading to its increased demand include the global demand for its recreational and medicinal use, as well as its reputation as an aphrodisiac. Our case report on mad honey disease aims to raise awareness of this condition, highlight its clinical presentation and management, and emphasize the possibility of its occurrence outside endemic regions.

**Case Presentation:** A 40-year-old Nepalese male living in Qatar presented with dizziness, hypotension, and severe bradycardia a few hours after consuming approximately 50 g of imported mad honey from Nepal. His admission ECG revealed sinus bradycardia without evidence of heart block. Initial stabilization was achieved with 0.5 mg of atropine and a norepinephrine infusion. The patient's symptoms resolved with supportive care while he was closely observed in the intensive care unit. He was discharged symptom free after 24 h.

**Conclusion:** This case, to the best of our knowledge, represents the first reported incidence of mad honey disease in Qatar, emphasizing the importance of recognizing this rare condition in nonendemic areas. Proper history-taking, particularly with a focus on food and ingestion history, along with a high index of clinical suspicion, is crucial for timely diagnosis and management. While unintentional and accidental overdose and poisoning, as occurred in our case, may happen sporadically, the widespread use and import/export of mad honey necessitates stringent measures and precautions, similar to those adopted by various countries.

## 1. Introduction

Mad honey disease is an uncommon but possibly lethal condition caused by consuming honey that contains grayanotoxins, which are neurotoxic substances present in the nectar of specific Rhododendron species. Although it is endemic to areas like the Black Sea region of Türkiye and the Himalayas, globalization and a growing demand for natural remedies and exotic foods have resulted in cases appearing in nontraditional locations. This case report details the first known occurrence of mad honey poisoning in Qatar, affecting a 40-year-old Nepalese expatriate who experienced severe bradycardia and hypotension after consuming imported mad honey from Nepal.

Since it often resembles other cardiovascular and neurological disorders, mad honey poisoning poses a diagnostic challenge. This is especially true in areas where it is not common. A comprehensive patient history, which encompasses dietary intake, and a strong clinical suspicion are crucial for timely recognition. This case highlights the significance of including mad honey toxicity in the differential diagnosis of unexplained bradycardia and hypotension, even in regions where it is not endemic. In addition, it underscores the wider ramifications of honey imports that lack regulation and the necessity for public health initiatives to reduce risks linked to honey consumption.

This report seeks to improve awareness among healthcare providers, aid in early intervention, and add to the expanding literature on mad honey poisoning in atypical geographic locations by detailing the patient's clinical course, diagnostic approach, and management. The results also support the idea of stricter regulatory oversight to avert similar incidents, especially in multicultural societies with varied dietary practices.

## 2. Case Report

A 40-year-old Nepalese male, working as a security guard, with no significant past medical history and no known allergies, was brought to the Emergency Department (ED) after his roommate called emergency medical services (EMSs). The EMS team found the patient to be bradycardic, with a heart rate below 30 bpm, and hypotensive, with a blood pressure of 93/61 mmHg.

Upon patient history taking, the patient stated that he had finished his night duty and then went to his friend's room. His friend offered him a type of natural honey that he had brought from Nepal, which the patient had never consumed before. At approximately 01:00 AM, he ingested around 50 g of what was later identified as mad honey and then went to bed. Around 04:00 AM, he woke up feeling unwell. When he went to the toilet, he experienced dizziness and nearly fainted. He managed to call for help, and his roommate immediately contacted EMS. His friend had also consumed some of the mad honey but did not develop any symptoms. According to the patient, his friend was accustomed to consuming this type of honey regularly.

On initial assessment by EMS, the patient was conscious and oriented. The patient had no skin rashes. He was bradycardic, with a heart rate of 30 bpm, and his blood pressure was 93/61 mmHg. His respiratory rate was 18 per minute while oxygen saturation was 98% on air. His heart sounds were normal, with no additional murmurs or abnormal findings. His chest and abdominal examinations were unremarkable. Notably, there was no wheeze or stridor. Neurological examination showed a Glasgow Coma Scale (GCS) of 15/15, with no focal neurological deficits. He was started on atropine 0.5 mg, noradrenaline, and intravenous fluids before being transported to the hospital. His heart rate and blood pressure showed initial improvement.

**Figure 1:**
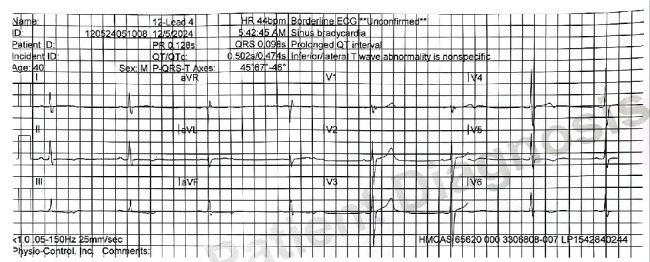
ECG showing bradycardia, HR 44.

A 12-lead ECG showed sinus bradycardia with no signs of heart block (Figure 1). The PR interval was 160 ms, within established normal limits. The QTc interval measured 470 ms, at the upper threshold of normal but not meeting criteria for prolongation. There were no significant ST segment or T wave changes.

Upon arrival at the ED at 05:00 AM, the patient's blood pressure was 150/99 mmHg, and his pulse rate was 94 bpm. The norepinephrine infusion was discontinued, and he was maintained on a continuous 0.9% normal saline infusion. His laboratory tests were unremarkable with normal CRP and WBCs. Notably his cardiac biomarkers including Trop T (5 ng/L) and Pro-BNP (12 pg/mL) were within normal reference limits. His ethanol level was also unremarkable (< 2.2 mmol/L). His Serum electrolytes—sodium, potassium, corrected calcium, magnesium, and phosphorus—were also within normal parameters.

In view of his history of mad honey ingestion, along with the initial symptoms and presentation of bradycardia and hypotension, a clinical diagnosis of mad honey syndrome was made. The diagnosis was based on a characteristic constellation of features and a compelling temporal association. The patient presented with profound bradycardia and hypotension within a few hours of ingesting a significant quantity of honey obtained from Nepal—a region where mad honey, known to contain grayanotoxins, is traditionally harvested and consumed. He had no prior medical history, was on no medications, and there was no reported exposure to toxins or recreational drugs. He was closely observed. At 07:40 AM, his blood pressure dropped again to 90/56 mmHg, and his pulse rate decreased to 45 bpm. He was given 0.5 mg IV atropine and started on a Ringer's lactate IV infusion before being admitted to the ICU for observation.

In the ICU, the patient showed gradual recovery, with improvement in both heart rate and blood pressure. After 24 h of observation, he was transferred to the medical ward. As he remained stable, he was discharged from the hospital within the next 24 h, with follow-up scheduled at the Internal Medicine Clinic and was strongly advised to avoid consuming mad honey in the future.

## 3. Discussion

To the best of our knowledge, at the time of writing, this is the first reported case of mad honey poisoning in Qatar. A literature search was conducted using PubMed and Google Scholar with the keywords: “mad honey,” “grayanotoxin,” “mad honey poisoning,” AND “Qatar.” No previously published cases from Qatar were identified at the time of writing.

Natural honey is among the most popular natural products consumed by humans for centuries. In addition to its nutritional value, including its vitamin and mineral content, it also possesses antioxidant, anti-inflammatory, and antimicrobial properties [1]. Besides being a nutrient and a natural sweetener, it has been used as a traditional medicine in many cultures.

A type of honey called “Mad Honey,” also known locally in Türkiye as “Deli Bal,” is distinct for its hallucinogenic effects. Unlike traditional honey, it is contaminated with a neurotoxin called grayanotoxane, which is produced by certain plants of the *Rhododendron* species (such as *Rhododendron ponticum* and *Rhododendron luteum*) [2]. These plants grow naturally in the Himalayan region from Nepal, the Black Sea region of Türkiye [3], and various parts of North America, Brazil, and Europe [4]. Honey made from the nectar of these plants contains grayanotoxanes, a group of neurotoxins that interfere with voltage-gated sodium channels. These toxins bind to the channels, preventing their inactivation and leading to prolonged depolarization of cells, which results in a variety of dose-dependent clinical effects.

When consumed in small quantities, mad honey can cause symptoms such as dizziness, hypotension, and bradycardia. In larger quantities, it may lead to more severe effects, including loss of consciousness, syncope, and atrioventricular block [5].

The use and demand for mad honey has increased over time. In addition to its use in traditional and alternative medicine due to its perceived health benefits, it is also sought for its psychoactive properties as a recreational substance and as an aphrodisiac. Some tourists, who enjoy experiencing new practices and foods, consume mad honey as part of their travel and cultural experiences. In addition, extensive global travel among diverse expatriate communities may contribute to its consumption sometimes without awareness of its harmful effects, as occurred in our case. The availability of mad honey through online platforms has introduced it to a global audience and facilitated worldwide consumption.

Since mad honey consumption can lead to serious health consequences, many countries have stringent regulations and restrictions on their sales and imports. In some cases, countries have completely banned their imports.

Mad honey disease is a rare condition, and its exact global prevalence is not well documented. However, the majority of reported cases occur in regions where toxic honey is produced, most notably in Türkiye [6]. To our knowledge, this is the first reported case of mad honey disease in Qatar, resulting from accidental ingestion. Diagnosing this condition in nonendemic regions is challenging due to its low incidence and limited awareness. However, timely recognition and appropriate management are crucial, as early intervention not only optimizes patient care but also reduces the need for extensive and unnecessary diagnostic investigations.

The severity of intoxication depends on both the amount of mad honey consumed and the concentration of grayanotoxanes in the honey. Yılmaz et al. reported in their case series of 66 patients that the amount of honey causing poisoning ranged from 5 to 30 g [7]. In a pooled analysis of 69 patients drawn from 11 studies and reports, as detailed by Gunduz et al. [3], 54% of individuals presented with sinus bradycardia, while 18.8% exhibited nonspecified bradyarrhythmia. Complete atrioventricular (AV) block was documented in nearly 9% of cases, and asystole occurred in 1.45%. The majority of patients had ingested between 5 and 30 g of honey. Our patient consumed approximately 50 g of honey. Symptoms typically appear after a dose-dependent latent period, ranging from a few minutes to two or more hours. Patients present with a wide variety of symptoms, including nausea, vomiting, dizziness, impaired consciousness, and cardiac arrhythmia. Sibel et al., in their systematic review of over 1000 cases, found that the most common symptoms of mad honey poisoning included dizziness, low pulse rate, nausea, vomiting, presyncope, and blurred vision [8]. The most critical parameters in clinical diagnosis were bradycardia and hypotension.

Although toxicological analysis to detect grayanotoxins in the honey sample was not available and, therefore, not performed, the diagnosis was supported by the exclusion of alternative causes. Specifically, beta-blocker overdose, organophosphate poisoning, and vasovagal syncope were considered and deemed unlikely, based on clinical history, physical examination, and laboratory results. There was no history of beta-blocker use, prescription medications, or overdose, and the patient denied any access to or ingestion of pharmaceuticals, making beta-blocker toxicity improbable. Furthermore, typical features of organophosphate poisoning—including miosis, excessive salivation, bronchorrhea, lacrimation, diarrhea, fasciculations, or altered mental status—were notably absent, and there was no known exposure to agricultural chemicals or pesticides. Vasovagal syncope was also considered but is unlikely given the absence of classic prodromal symptoms (such as nausea, pallor, or diaphoresis), the presence of significant bradycardia and hypotension persisting beyond a brief syncopal event, and the lack of clear situational triggers such as emotional distress or prolonged standing. The temporal association with mad honey ingestion, along with the typical presentation of hypotension and bradycardia, supports the diagnosis of mad honey poisoning, which is consistent with the known physiological effects of grayanotoxin. The patient's rapid improvement with supportive therapy and atropine—without the need for specific antidotes—further supports the diagnosis. While we recognize that confirmatory toxin testing was not undertaken, the clinical presentation aligns closely with reported cases of mad honey poisoning, and this limitation is acknowledged.

Although ingestion of 1–5 spoons of mad honey has been associated with intoxication effects, prolonged consumption has been shown to induce desensitization of sodium channels in excitable cells. In this case, the patient exhibited severe symptoms following mad honey consumption, whereas his friend, who had seemingly been consuming it regularly, did not experience significant symptoms. This observation suggests a likely adaptive mechanism and tolerance development in habitual users. Management of mad honey disease includes IV fluids to maintain adequate hydration and hemodynamic stability, especially in cases of hypotension. For symptomatic bradycardia, IV atropine is the first-line medication, and vasopressors are used in cases of severe hypotension refractory to fluids and atropine. Most patients recover within 24–48 h with supportive care.

## 4. Conclusion

This case, to the best of our knowledge, represents the first reported incidence of mad honey disease in Qatar, emphasizing the importance of recognizing this rare condition in nonendemic areas. Proper history-taking, particularly with a focus on food and ingestion history, along with a high index of clinical suspicion, is crucial for timely diagnosis and management. While unintentional and accidental overdose and poisoning, as occurred in our case, may happen sporadically, the widespread use and import/export of mad honey necessitates stringent measures and precautions, similar to those adopted by various countries.

## Data Availability

The data that support the findings of this study are available on request from the corresponding author.
